# Activation of dopaminergic VTA inputs to the mPFC ameliorates chronic stress‐induced breast tumor progression

**DOI:** 10.1111/cns.13465

**Published:** 2020-10-28

**Authors:** Xi‐Rong Xu, Qian Xiao, Yu‐Chuan Hong, Yun‐Hui Liu, Yue Liu, Jie Tu

**Affiliations:** ^1^ The Brain Cognition and Brain Disease Institute (BCBDI), CAS Key Laboratory of Brain Connectome and Manipulation, Shenzhen Institutes of Advanced Technology Shenzhen‐Hong Kong Institute of Brain Science‐Shenzhen Fundamental Research Institutions Chinese Academy of Sciences Shenzhen China; ^2^ University of Chinese of Academy of Sciences Beijing China; ^3^ Center of Brain Science, State Key Laboratory of Magnetic Resonance and Atomic and Molecular Physics National Center for Magnetic Resonance in Wuhan Key Laboratory of Magnetic Resonance in Biological Systems, Wuhan Institute of Physics and Mathematics Innovation Academy for Precision Measurement Science and Technology Chinese Academy of Sciences Wuhan China

**Keywords:** cancer, emotion, neuromodulation, neurotransmitter, optogenetic, unpredictable mild stress

## Abstract

**Aims:**

Chronic stress plays an important role in promoting the progression and migration of cancers. However, little is known of any direct impact on tumor progression related to the regulation of emotion‐related circuitry. The aim of this study was to explore the neural‐circuit mechanisms underlying stress‐induced progression of cancers and the impact of emotion‐related regulation of circuitry on tumor growth.

**Methods:**

Optogenetic manipulation was applied to unpredictable chronic mild stress (UCMS)–treated mice bearing breast tumor cell. The stress‐related hormones, tumor‐related cytokines, the tyrosine hydroxylase (TH)–positive neurons and their fibers, dopamine receptor–positive cells, and anxiety level were measured using ELISA, immunohistochemical staining, fluorescence in situ hybridization, and behavioral test, respectively.

**Results:**

By investigating breast cancer mouse models with a chronic mild stress model, optogenetic stimulation, and behavioral analysis, we show that chronic stress induced anxiety‐like behavior in mice and increased serum concentration of norepinephrine and corticosterone, hormones closely related to stress and anxiety. Optogenetic activation of VTA TH terminals in the mPFC rescued anxiety‐like behavior induced by chronic stress. Chronic stress resulted in marked progression of breast tumors, and repetitive optogenetic activation of VTA TH terminals in the mPFC significantly attenuated stress‐induced progression of breast cancers and reduced serum concentration of norepinephrine and corticosterone. Furthermore, there was a positive correlation between serum norepinephrine or corticosterone concentration and tumor size.

**Conclusions:**

These findings indicate a positive role of an emotion regulation circuit on the progression of breast cancer and reveal a link between stress, emotion regulation, and the progression of breast cancers. Our findings provide new insights pertinent to therapeutic interventions in the treatment of breast cancers.

## Graphical abstract

Chronic stressors resulted in marked progression of breast tumors. Repetitive activation of the VTA^TH^ terminals within the mPFC by optogenetic manipulation significantly attenuated stress‐induced progression of breast tumors

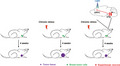

## 1 INTRODUCTION

Exposure to stress is an important risk factor for psychiatric disorders, such as anxiety and depression.^1^, ^2^ One patient population that typically live with chronic stress is cancer patients. Deleterious consequences include stress‐induced psychiatric disorders, which adversely affect the progression of cancers and treatment outcomes.^3^, ^4^, ^5^, ^6^, ^7^ Chronic stress markedly affects the incidence and progression of many kinds of cancers in both human and animal studies.^5^, ^8^, ^9^, ^10^ Despite the evidence that cancer progression is differentially modulated by stress‐related disorders through various mechanisms and conditions, little is known about the neural‐circuit mechanisms underlying stress‐induced progression of cancers and the impact of emotion‐related regulation of circuitry on tumor growth.

The medial prefrontal cortex (mPFC) plays a critical role in regulating responses to stress.^11^ A large body of evidence has shown that the mPFC receives dopaminergic afferents emanating from the ventral tegmental area (VTA) and that this pathway is critical in neural circuits that have been implicated in the regulation of emotion,^12^ cognition and memory,^13^ and attentional processes.^14^ Study using mice has shown that optogenetic stimulation of dopamine receptor 1 (D1) cells in the mPFC has an antidepressant effect and infusion of a D1 receptor agonist into the mPFC produces antidepressant symptoms.^12^ Furthermore, dopamine (DA), which is a stress‐related hormone, is an important part of an adaptive response to chronic stress^15^ through the regulation of tumor progression and cancer cell proliferation through dopamine receptors.^16^, ^17^, ^18^ However, whether the activation of DA neurons within the VTA‐mPFC structure plays a role in the regulation of tumor progression has not been determined.

In this study, we investigated the neural‐circuit mechanism underlying stress‐induced progression of cancers. To study the role of chronic stress on cancer progression, an unpredictable chronic mild stress (UCMS) mouse model protocol was used and anxiety levels were evaluated using behavioral analysis and detection of stress‐related hormones. The UCMS model was then used to determine the effect of stress on tumor progression in nude mice bearing breast tumor cells. To investigate the impact of emotional regulation by activation of a neural circuit on tumor growth, we injected AAV‐TH‐Cre and AAV‐DIO‐ChR2/mCherry viruses into the mouse VTA and implanted optic fibers into the mPFC. This approach allowed the selective activation of dopaminergic VTA neurons projecting to the mPFC. Acute stimulation of the VTA^TH^–mPFC circuit during behavioral tests was then used to verify the role of this dopaminergic projection in regulating emotional state. Chronic repetitive stimulation of this circuit was used to examine the relationship between chronic stress, reduced anxiety levels, and tumor progression. Using a combination of breast cancer mouse models, chronic repetitive optogenetic stimulation, and behavioral analysis, we reveal that breast cancer progression under anxious states was markedly increased. We also found that optogenetic stimulation of VTA^TH^ terminals within the mPFC rescued chronic stress‐induced anxiety‐like behavior and also significantly attenuated the increased progression of breast tumors.

Our data demonstrate a previously undescribed cell‐ and circuit‐level mechanism, which adds to our understanding of stress and the progression of the breast cancers.

## 2 MATERIALS AND METHODS

### 2.1 Animals

Male C57BL/6J mice (Charles River, Beijing, China) aged 6‐8 weeks (18‐22 g) and male BALB/c nude mice (Hunan Silaike Jingda Laboratory Animal Co Ltd, China) aged 6‐8 weeks (20‐25 g) were used. All mice were maintained in specific‐pathogen‐free conditions under a 12‐h light/12‐h dark cycle, with ad libitum access to food and water. Procedures were approved by the Institutional Animal Care and Use Committee of Shenzhen Institutes of Advanced Technology, Chinese Academy of Sciences, and all experimental procedures involving animals were carried out in strict accordance with the Guidelines for the Animal Care and Use Committee. Surgeries were performed under full anesthesia, and every effort was made to minimize animal suffering.

### 2.2 Unpredictable chronic mild stress procedure (UCMS)

The UCMS protocol was performed as previously described,^19^ with minor modifications. The stress group was randomly assigned to one of the following environmental stressors during each daily session for 4 weeks: (a) overnight illumination where mice were exposed to regular room light during the night period; (b) squeezing, where 8‐12 mice were placed in one box that was normally used to hold mice food for 2 hours; (c) wet bedding, where mice were placed inside a homecage on a pad that was suitably wet with drinking water for the duration of the night period; (d) restraint, where mice were placed for 1 hour in a 50‐mL plastic tube with both ends open to allow air; (e) inverted light cycle, where regular room light was off during the day and on during the night for 2 days, and (f) tilted cage, where homecages were tilted at a 45° angle for 2 hours. Bodyweight was monitored every three days.

### 2.3 Behavioral tests

For all animal experiments, littermate mice were randomly assigned to the experimental groups and were identifiable by a unique identification number. Experimenters were blind to experimental group allocation. In addition, data analysts were blind to experimental conditions. Groups of mice were age‐matched (8‐14 weeks). All mice were handled for 15‐30 minutes per day for three days prior to behavioral assays to reduce stress introduced by contact with experimenter. Behavioral tests were used to evaluate anxiety levels in mice.

#### 2.3.1 Elevated plus‐maze test (EPM)

A plastic elevated plus maze consisting of a central platform (5 × 5 cm) with two white open arms (25 × 5×25 cm) and two white closed arms (25 × 5×25 cm) extending from it in a plus shape was used. The maze was elevated 65 cm above the floor. Mice were individually placed in the center with their heads facing a closed arm. The number of entries and the amount of time spent in each arm type were recorded.

#### 2.3.2 Open‐field test (OFT)

A plastic open‐field square chamber (50 × 50 cm) was used and conceptually divided into a central field (25 × 25 cm) and a peripheral field for analysis. Each mouse was placed in a random corner of the arena at the start of each test. The number of entries and the amount of time spent in the center and corners were recorded, as was the total distance traveled. Locomotor and exploratory activity was assessed using the OFT. Distance traveled was used as a measure of locomotor activity.

### 2.4 In vitro culture of human and mouse breast cancer cell lines

Human breast cancer cell lines MCF‐7 and MDA‐MB‐23 and mouse breast cancer cell line 4T1 were used (Chinese Academy of Sciences Cell Bank). The MCF‐7, MDA‐MB‐231, and 4T1 cells were cultured separately in DMEM (HyClone, SH30243.01) supplemented with 10% fetal bovine serum (Gibco, 10099‐141) and 1% PS (penicillin and streptomycin sulfate; HyClone SV30010). Adherent monolayer cultures were maintained on plastic dishes or bottles and incubated at 37°C in a mixture of 5% CO_2_ and 95% air.

### 2.5 Tumor implantation

To generate animals with the breast cancer model induced by MDA‐231 and MCF‐7 breast cancer cells, BALB/c nude male mice received s.c. injections of 0.1 mL cell suspension containing 2 × 10^6^ cells to left flank areas. Briefly, cultured cancer cells were detached by trypsinization (Gibco, 25200072), collected by centrifugation, resuspended in medium, and the cell suspension was then injected s.c. into the 8‐ to 10‐week‐old BALB/c nude male mice. When tumors became measurable, tumor volume was measured once weekly. Tumor volume was calculated using the formula, tumor volume (mm^3^) = 0.5 × a × b^2^, where a was the largest diameter and b was the perpendicular diameter.^20^

### 2.6 Cell viability assay

Cell viability was analyzed by Cell Counting Kit‐8 assay (TransGen Biotech, Beijing, China). Cells (8 × 10^3^) were dissociated and seeded in 96‐well plates at a density of 8 × 10^3^/well, cultured for 24 hours, and then removed from the medium and treated with fresh medium including dopamine hydrochloride (Sigma, H8502) with 0, 0.5, 1.0, 2.0, and 4.0 μmol. After incubation for 24 hours, cells were cultured with CKK solution for 2 hours after removing the medium. The absorbance value of 450 nm was detected using a microplate reader (Infinite 200PRO) 2 hours later.

### 2.7 Hematoxylin and eosin staining

Hematoxylin and eosin (H&E) staining was conducted according to a routine procedure.^21^ Briefly, after fixation and dehydration, freshly collected breast tumor tissues were made into 5‐μm paraffin sections for H&E staining. After deparaffinization and rehydration, tumor sections were stained with hematoxylin solution for 5 minutes, rinsed in running tap water for 5‐10 seconds followed by differentiation using 0.1% hydrochloric acid‐ethanol for 1‐3 seconds, and then rinsed in running tap water for 10‐30 seconds. Then, the sections were blued for 10‐30 seconds and rinsed in running tap water for 10‐15 minutes. Next, the sections were stained with eosin solution for 1‐3 minutes followed by dehydration with graded alcohol and clearing in xylene. The mounted slides were then examined and photographed using a normal fluorescence microscope (Axio Scope.A1, ZEISS).

### 2.8 Surgery and virus injection

For all stereotaxic surgeries, animals were anesthetized with 1% pentobarbital sodium (20 mg/kg) prior to surgery. Once anesthetized, hair at the incision site was removed and eyes were coated with ophthalmic ointment. Next, the animals were head‐fixed in a stereotaxic apparatus (RWD, China) and the incision site was sterilized. Craniotomies were made, and a Hamilton syringe (Neuros; Hamilton, Reno, USA) fitted with a 33‐gauge needle was used to place a viral bolus (250 nl) at the following coordinates within the lateral VTA (AP: −3.4 mm, ML: 0.3 mm, DV: −4.5 mm). Optical fibers (200 μm in diameter, NA: 0.37, RWD, China) were implanted in the mPFC (AP: 1.9 mm, ML: 0.4 mm, DV: −2.6 mm). Cannula was fixed to the skull using dental cement and a pair of skull screws.

We obtained AAV‐TH‐Cre (rAAV‐TH‐NLS‐Cre‐WPRE‐pA), AAV‐DIO‐ChR2‐mCherry (rAAV‐EF1a‐DIO‐hChR2‐mCherry), and AAV‐DIO‐mCherry (rAAV‐EF1a‐DIO‐mCherry) viruses (BrainVTA, China) for optogenetic experiments. A 250 nl virus cocktail (rAAV‐TH‐Cre and AAV‐DIO‐ChR2/mCherry) was injected into the lateral VTA (AP: −3.4 mm, ML: 0.3 mm, DV: −4.5 mm), and optical fibers were implanted unilaterally into the mPFC (AP: 1.9 mm, ML: 0.4 mm, DV: −2.6 mm). A control group was injected with a virus cocktail including AAV‐TH‐Cre and AAV‐DIO‐mCherry and received optogenetic stimulation but not the UCMS treatment. Mice in the stress‐mCherry group were injected with the same virus as the control group but also received UCMS treatment and optogenetic stimulation. This meant that the control and stress‐mCherry groups did not express the channelrhodopsin (ChR2), a light‐sensitive channel protein that can be used to activate neurons following optogenetic stimulation. Mice in the stress‐ChR2 group were injected with a virus cocktail including AAV‐TH‐Cre and AAV‐DIO‐ChR2‐mCherry and received UCMS treatment and optogenetic stimulation. Those mice implanted with an optical fiber in the mPFC were given a one‐week recovery period prior to experiments.

### 2.9 In vivo optogenetic behavioral tests and chronic repetitive stimulation

During in vivo optogenetic behavioral tests (Figure 4) in awake, freely moving mice, the optic fiber was connected to a laser source using an optic fiber sleeve. The control group underwent the same procedure and received the same intensity of laser stimulation (473 nm, 10 Hz, 15‐ms width, 5‐10 mW power for mPFC terminals, 5‐min OFF/5‐min ON × 1; no seizure‐like behavior observed during stimulations; n = 6‐8/group) as the s‐mCherry and s‐ChR2 groups. During chronic repetitive light stimulation (Figure 5), all nude mice groups received the same laser stimulation (10 Hz, 15‐ms, 5‐min ON/5‐min OFF × 3, 30 minutes) every second day during UCMS.

### 2.10 Immunohistochemistry

After completion of experiments, mice were deeply anesthetized with 10% chloral hydrate (400 mg/kg) and transcardially perfused with PBS, and then ice‐cold 4% paraformaldehyde (PFA) in PBS (wt/vol). Brains were dissected and postfixed at 4°C in 4% PFA overnight and then equilibrated in 30% sucrose in PBS. Next, 30‐μm coronal slices were cut using a freezing microtome. Slices were stored in a cryoprotection solution at 4°C until processed further. Immunohistochemistry was performed to map the co‐localization of ChR2‐mCherry virus expression in the VTA with TH immunoreactivity. Antibody staining was performed on single‐well floating tissue sections. Sections were incubated overnight in primary antibodies at 4°C followed by 1‐h incubation with secondary antibodies at room temperature (RT). The primary antibody used in this study was anti‐tyrosine hydroxylase antibody (Millipore, MAB318; 1:200). Suitable secondary antibodies were chosen to reveal the co‐localization with different fluorescent colors. For counterstaining, sections were incubated for 5 minutes with 4’, 6‐diamidino‐2‐phenylindol (DAPI, 0.4 mg/mL, Sigma). All images were captured with a Zeiss LSM880 confocal microscope.

### 2.11 Fluorescence in situ hybridization

We used single‐probe in situ hybridization (ISH) on fixed‐frozen tumor tissue sections. For single probes, coding region fragments of D1 and D2 receptors were isolated from tissue cDNA using PCR and cloned into the pCR4 Topo Vector (Thermo Fisher). Digoxigenin (DIG)‐labeled riboprobes were prepared using a DIG RNA Labeling Kit (11277073910, Roche). Tumor tissue sections were hybridized with DIG‐labeled cRNA probes at 56°C for 14‐16 hours. After hybridization, sections were washed twice in 0.2 × SSC at 65°C for 20 minutes and then incubated with horseradish peroxidase (POD)‐conjugated sheep anti‐DIG antibodies (1:300; 1207733910, Roche) diluted in blocking buffer (1% Blocking reagent, FP1012, Perkin Elmer) for 45 minutes at RT. Sections were washed three times for 5 minutes at RT in PBST (0.05% Tween 20 in PBS) wash buffer and then treated using a TSA Plus Cy5 Kit (1:100; NEL745001KT, Perkin Elmer) for 10 minutes at RT. Sections were mounted in Fluoromount‐G (0100‐20, Southern Biotech) and then imaged using an Zeiss Axio Imager Z2 microscope.

### 2.12 ELISA analysis

Fresh blood was collected and deposited at room temperature and centrifuged at 9000 *g* for collection of supernatants. The supernatants were stored at −20°C for later use. Murine serum norepinephrine, corticosterone, and VEGF were measured by NE (MyBioSource, MBS776673, and Abnova, KA1891), CORT (MyBioSource, MBS775946, and Abcam, ab108821), and VEGF (MyBioSource, MBS412303) ELISA Kits, respectively. Murine serum IL‐1a, IL‐6, and bFGF were measured by IL‐1a (Abcam, ab113344), IL‐6 (Abcam, ab100712), and bFGF ELISA Kits (Abcam, ab100670), respectively.

### 2.13 Statistics

Statistical analyses were conducted, and graphs made, using GraphPad Prism 7. All results are presented as the mean ± SEM of at least five parallel assessments (refer to detailed explications in figure legends). The statistical significance of group differences was evaluated by Student's *t* tests for the comparison of two groups or analysis of variances (ANOVA) followed by the Bonferroni post hoc test for the comparison of three and more groups. Kolmogorov‐Smirnov test was used to assess data distribution. When data followed a normal distribution but did not obey homogeneity of variance, Welch's *t* tests were used. When data did not follow a normal distribution and did not obey homogeneity of variance, the nonparametric Mann‐Whitney test for comparison of two groups and the Kruskal‐Wallis test for comparison of three or more groups were used to calculate the statistical significance of group differences. All statistical parameters for specific analyses are described in the appropriate figure legend. A value of *P* < .05 was considered statistically significant.

## 3 RESULTS

### 3.1 Chronic stressors induce anxiety‐like behavior in C57BL/6J (C57) mice

**FIGURE 1:**
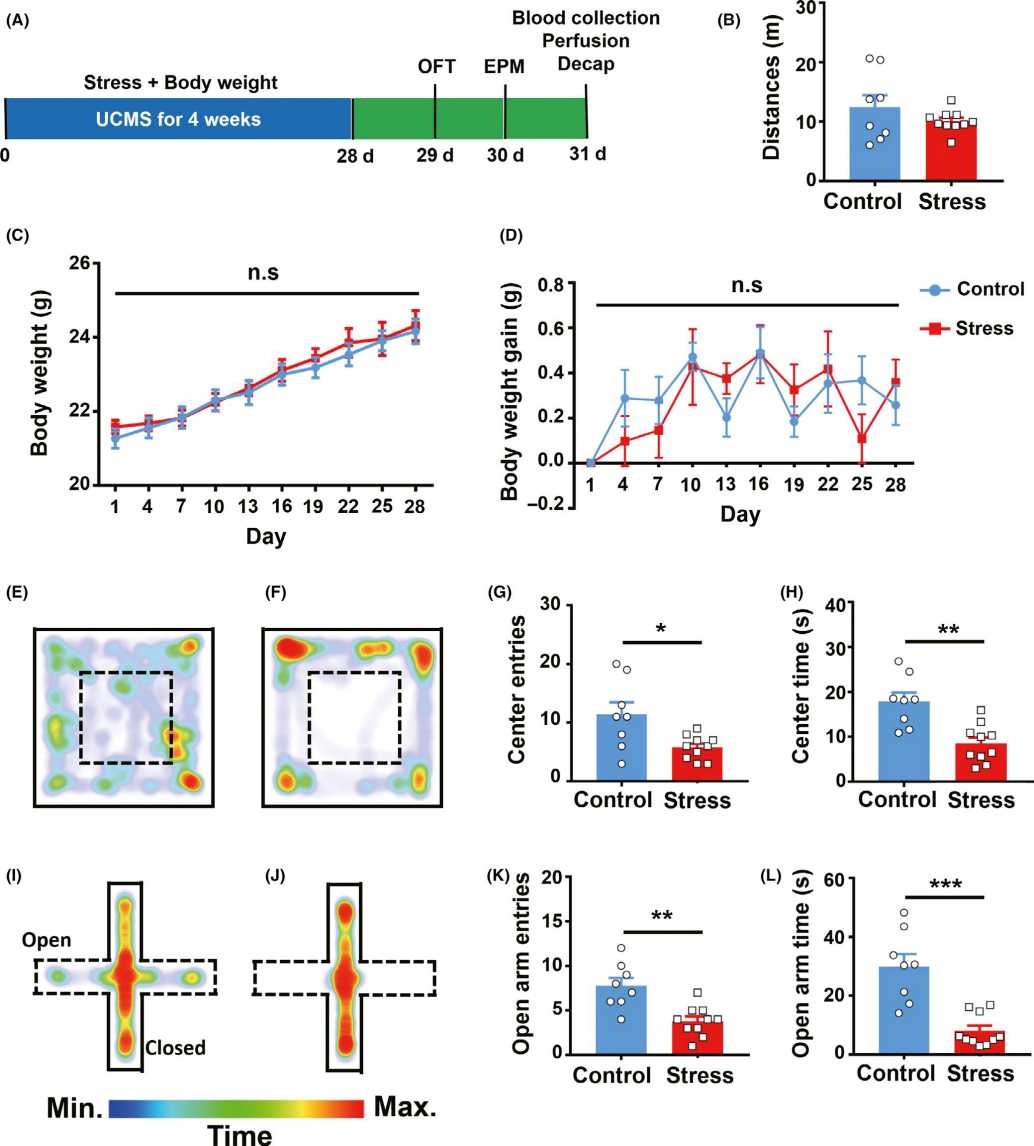
Chronic stress results in anxiety‐like behavior in C57BL6/J mice. (A), Schematic illustration of the chronic stress paradigm. (B), Locomotor activity in the control and stressed groups; n = 8 control, n = 10 stress, Welch's t test. (C) ~ (D), Bodyweight and bodyweight gain were recorded during 4 weeks of chronic stress. (E) ~ (F), Heat maps of control and stressed mice in different positions on the open field (OF). Warm color indicates high time spent at that location. (G) ~ (H), Entries to the OF center and time in the OF center; n = 8 control, n = 10 stress, G, Welch's t test, H, unpaired t test. (I) ~ (J), Heat maps of control and stressed mice in different positions in the elevated plus maze. (K) ~ (L), Entries to the open arms of the elevated plus maze (EPM) and time on the open arms of the EPM; n = 8 control, n = 10 stress, K, unpaired t test, L, Mann‐Whitney test, *P < .05, **P < .01, ***P < .001

We adopted a chronic stress model to investigate the impact of UCMS stressors on the regulation of bodyweight and emotional behavior, such as anxiety. Two groups of C57 mice, (a) standard diet without stressors (control) and (b) standard diet with stressors (stress), were compared in these experiments (Figure 1A). We found no significant difference in locomotor activity (Figure 1B). Neither was there a significant difference in bodyweight (Figure 1C) or bodyweight gain (Figure 1D) between the control and stress groups throughout the 4‐week period. These results indicate that chronic stressors did not affect bodyweight or bodyweight gain, which is consistent with previous work.^22^ After exposure to chronic stressors, mice showed higher anxious states demonstrated by markedly lower exploration of the center in the OFT and open arms of the EPM (Figure 1E–L) when compared to their control littermates. These data indicate that chronic stressors induced anxiety‐like behavior.

### 3.2 Chronic stressors increase stress‐related hormones in both in BLAB/c nude mice and in C57 mice

**FIGURE 2:**
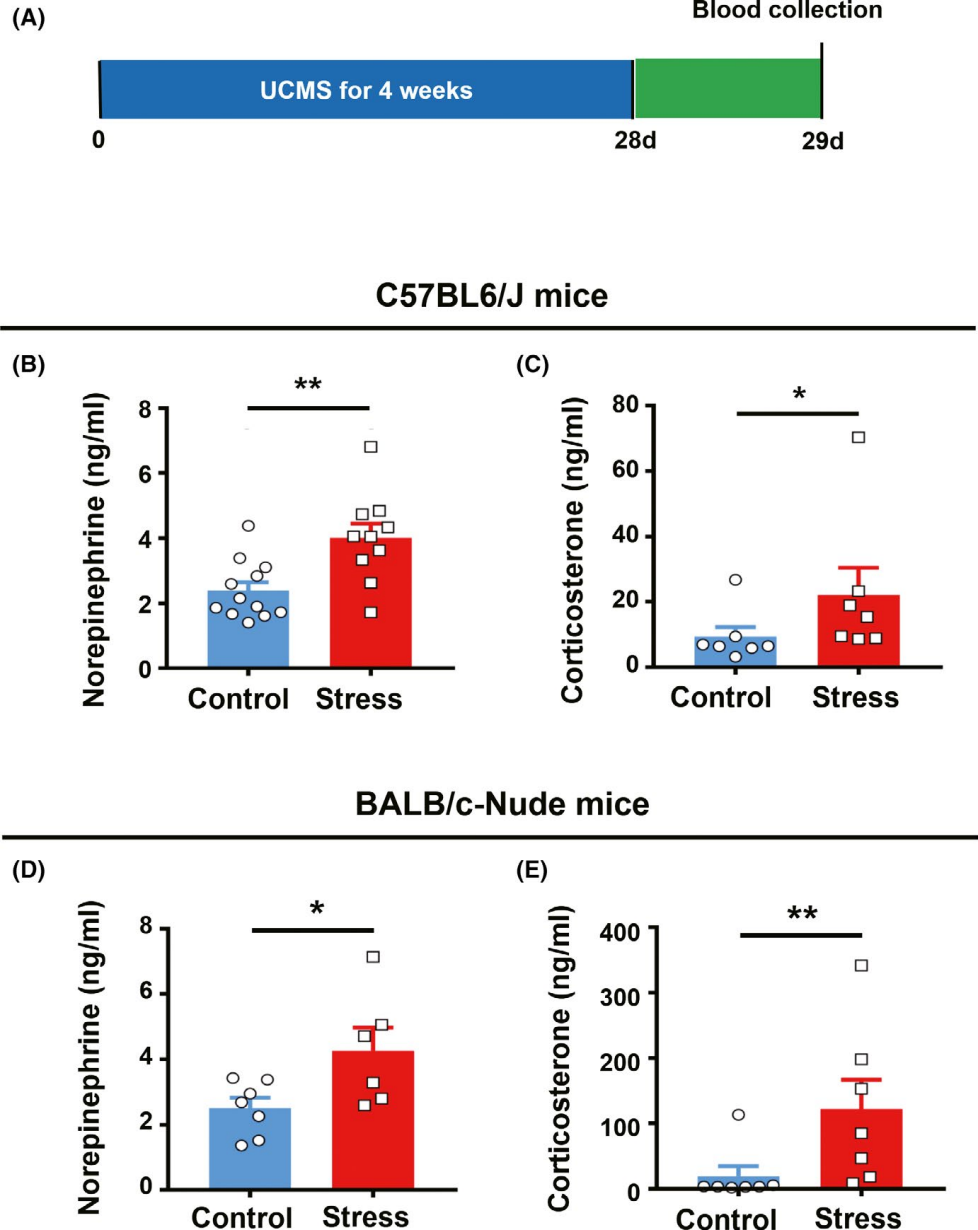
Chronic stress induces an increase in corticosterone and norepinephrine in both C57BL6/J and BALB/c nude mice. (A), Schematic illustration of the experiment design. (B), Serum NE concentrations from the control and stressed groups in C57BL6/J mice; n = 12 control, n = 10 stress, unpaired t test. (C), Serum CORT concentrations in control and stressed C57BL6/J mice; n = 7 control, n = 7 stress; Mann‐Whitney test. (D), Serum NE concentrations from control and stressed groups in BALB/c nude mice; n = 7 control, n = 6 stress; unpaired t test. (E), Serum CORT concentrations in control and stressed BALB/c nude mice; n = 7 control, n = 7 stress, Mann‐Whitney test. *P < .05, **P < .01, ***P < .001

The stress‐related hormones corticosterone (CORT) and norepinephrine (NE) were both markedly higher in the serum of stressed mice than in controls (Figure 2B‐C). We also found that both CORT and NE were significantly higher in stressed nude mice than in naive nude control mice (Figure 2D‐E).

### 3.3 Chronic stressors induce loss of bodyweight and accelerate tumor progression

**FIGURE 3:**
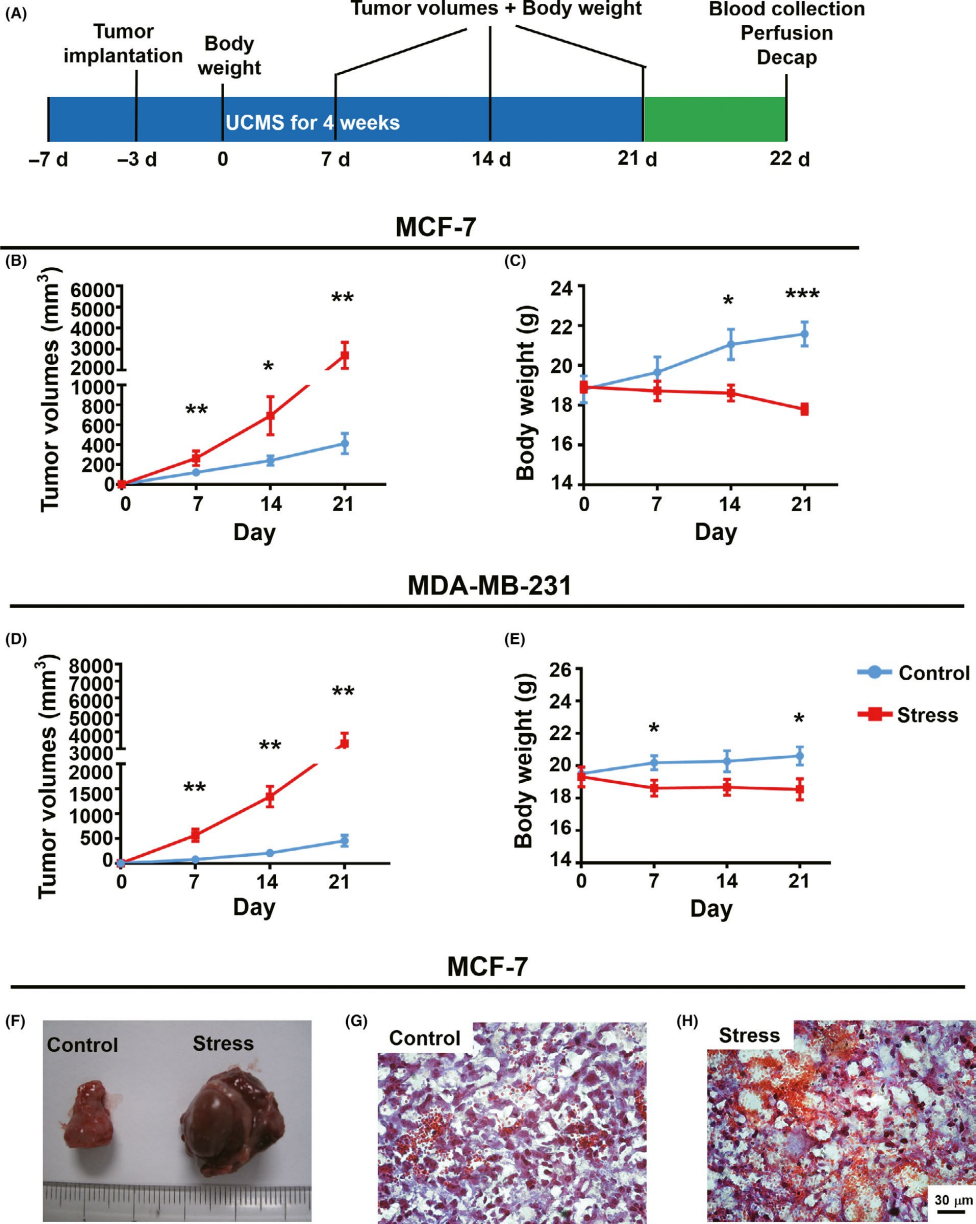
Chronic stress induces marked progression of breast tumor in BALB/c nude mice. (A), Schematic illustration of the experiment design. (B), Tumor volumes in MCF‐7 tumor‐bearing nude mice with and without exposure to chronic stressors; n = 7 control, n = 7 stress, Mann‐Whitney test for day 7, Welch's t test for days 14 and 21. (C), Bodyweight changes in mice bearing MCF‐7 with and without exposure to chronic stressors; n = 7 control, n = 7 stress, unpaired t test. (D), Tumor volumes in mice bearing MDA‐MB‐231 tumors with and without exposure to chronic stressors; n = 6 control, n = 6 stress, Mann‐Whitney test for day 14, Welch's t test for days 7 and 14. (E), Bodyweight in nude mice bearing MDA‐MB‐231 tumors with and without exposure to chronic stressors; n = 6 control, n = 6 stress, unpaired t test. (F), Representative images of tumors from nude mice bearing MCF‐7 tumors with and without exposure to chronic stressors. (G) ~ (H), Representative images of MCF‐7 breast cancer tissue with and without exposure to chronic stressors. Scale bar, 30 μm. *P < .05, **P < .01, ***P < .001

To investigate whether UCMS promotes the development of breast cancer, we measured tumor volume in two breast cancer models with or without stressors (Figure 3A). The tumor volume of stressed mice bearing MCF‐7 or MDA‐231 breast cancer cells was significantly larger than control tumor mice (Figure 3B‐E). Hematoxylin and eosin staining showed that chronic stressors facilitated the formation of breast cancer tumors (Figure 3G‐H). However, bodyweight in the stressed nude mice ended up significantly lower than that of the control group (Figure 3C‐E), whereas in C57BL6/J mice, bodyweight was not affected by daily UCMS over the 4‐week period (Figure 1C‐D). This suggests that tumor progression may affect food intake and energy metabolism in nude mice resulting in bodyweight reduction. Together, these data indicate that daily UCMS for a period of 4 weeks promoted breast tumor growth and development.

### 3.4 Optogenetic stimulation of VTA^TH^ inputs to mPFC rescues anxiety‐like behavior induced by chronic stressors

**FIGURE 4:**
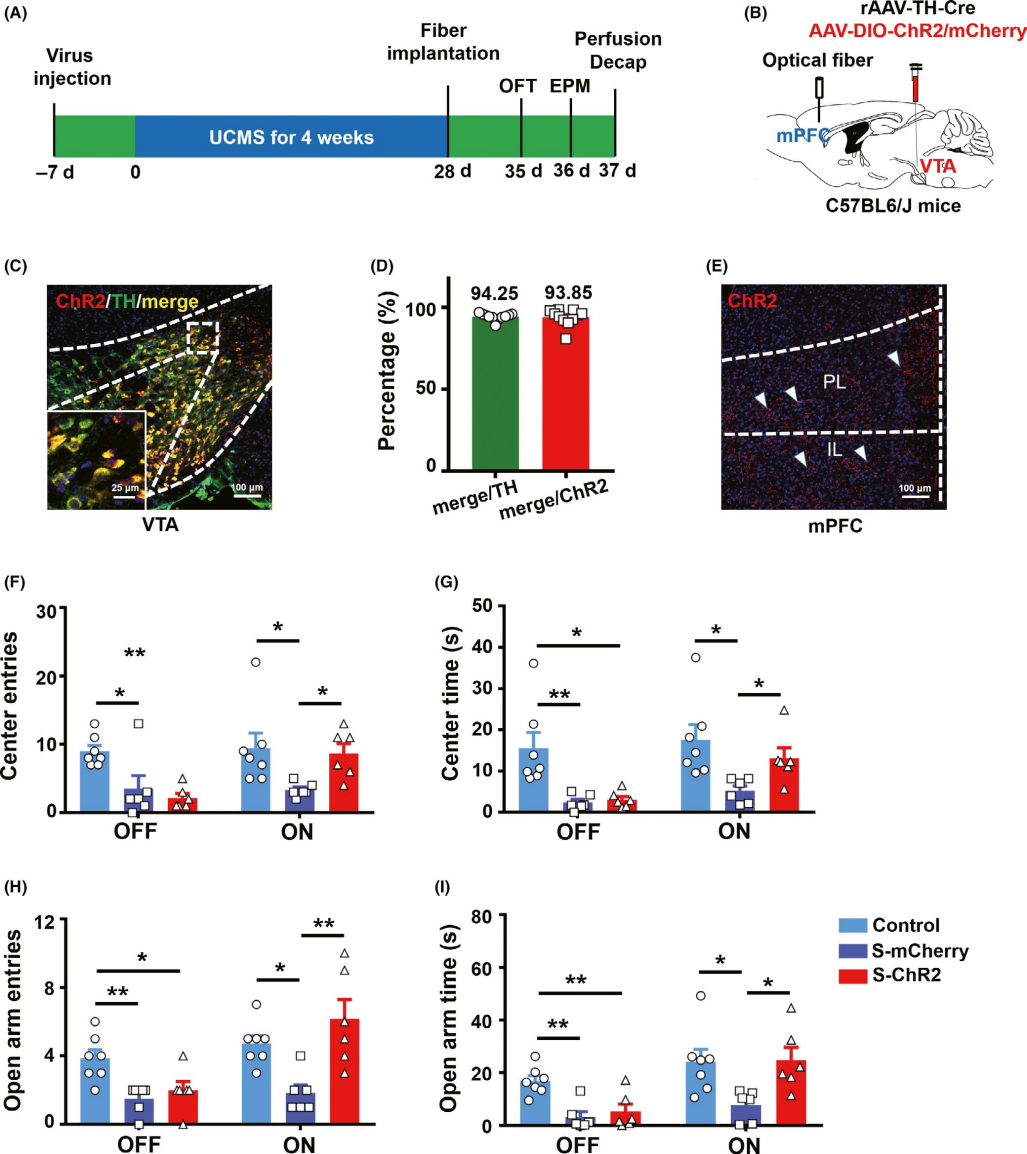
Optogenetic stimulation of VTA^TH^ terminals in the mPFC ameliorates anxiety‐like behavior induced by chronic stress in C57BL6/J mice. (A) ~ (B), Schematic illustration of the optogenetic experiment design. (C), Coronal image showing co‐localization of ChR2‐mCherry with TH‐expressing neurons in the VTA of C57BL6/J mice. Red, ChR2‐mCherry; green, TH‐positive neurons. Scale bar, 100 μm, insert scale bar, 25 μm. (D), Quantification of TH or ChR2‐mCherry neurons in the VTA, n = 11 slices from 3 mice. (E), Representative images showing the distribution of ChR2‐mCherry‐expressing terminals in the mPFC. Red: ChR2‐mCherry. Scale bar, 100 μm. (F) ~ (G), Entries to the OF center and time in the OF center; n = 7 control, n = 6 S‐mCherry, n = 6 mice S‐ChR2, ANOVA, Bonferroni post hoc test. (H) ~ (I), Entries to the open arms of the EPM and time on the open arms of the EPM; n = 7 control, n = 6 S‐mCherry, n = 6 S‐ChR2, ANOVA, Bonferroni post hoc test. *P < .05, **P < .01, ***P < .001

To investigate the involvement of VTA‐mPFC in the regulation of anxiety‐like behavior induced by chronic stressors, we optogenetically activated VTA^TH^ terminals in the mPFC of C57 mice (Figure 4A‐B).

Immunostaining revealed that 94.95 ± 0.45% of TH‐immunopositive cells were positive for ChR2, and 96.06 ± 0.78% of ChR2‐expressing cells, were co‐labeled with TH (Figure 4C–D), which demonstrates that the virus was effectively expressed and stable in the VTA. Also, robust ChR2 terminal expression was found in the mPFC (Figure 4E). In the OFT, light stimulation of the VTA^TH^ inputs to the mPFC^23^ led to increased exploration of the center, indexed by more center entries and center time in the ChR2 group compared with mCherry controls (Figure 4F‐G). Similarly, in the EPM test, the ChR2 group had a higher number of open‐arm entries and time spent in open arms during light stimulation of the VTA^TH^ terminals in the mPFC than the mCherry controls. Taken together, these data indicate that optogenetic activation of VTA^TH^ inputs to the mPFC ameliorated the anxiety induced by chronic stress.

### 3.5 Chronic repetitive optogenetic stimulation of VTA^TH^ neuron inputs to the mPFC attenuates breast tumor progression induced by chronic stressors

**FIGURE 5:**
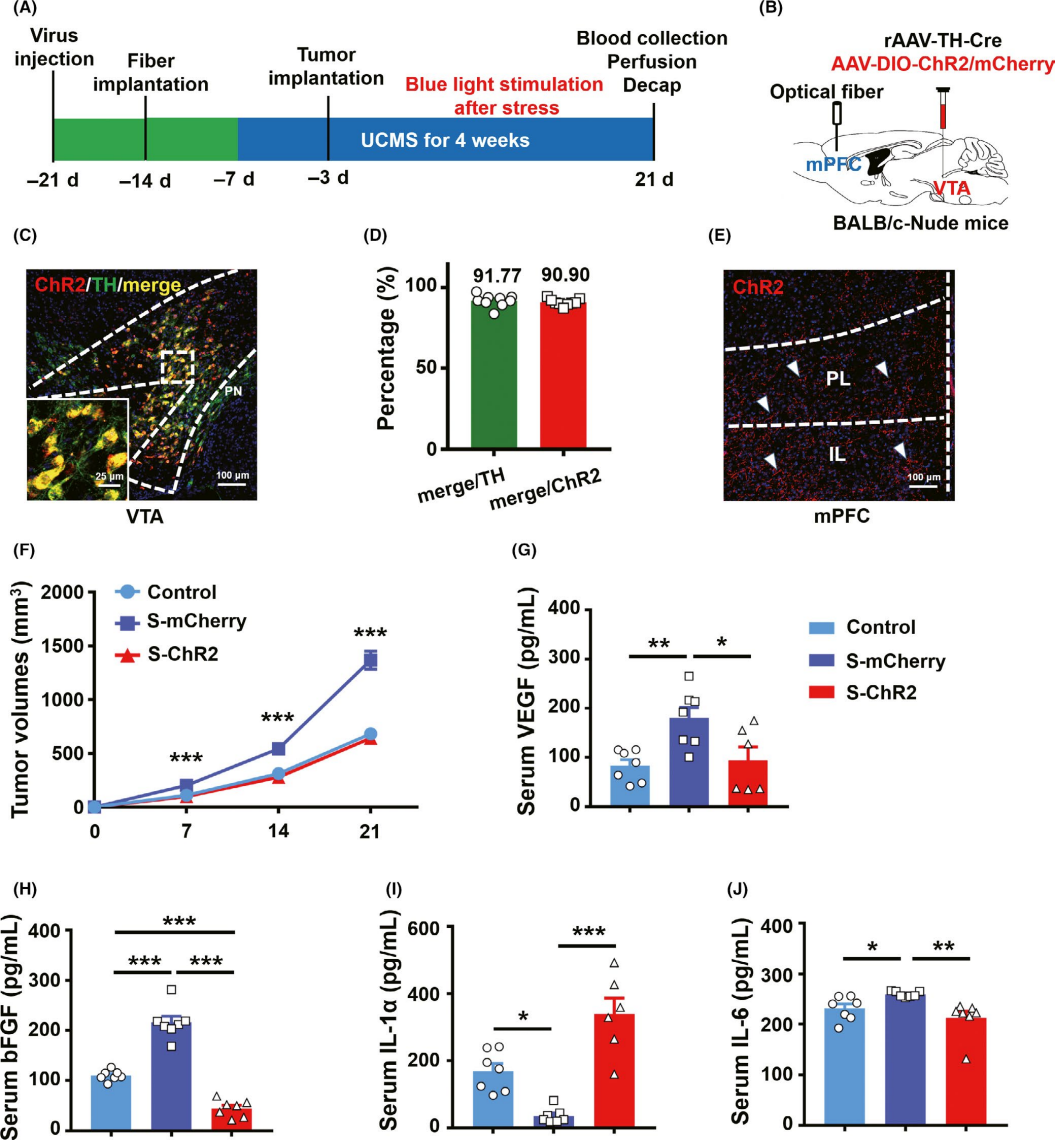
Repeated optogenetic stimulation of VTA^TH^ terminals in the mPFC ameliorates tumor progression induced by chronic stress in BALB/c nude mice. (A) ~ (B), Schematic illustration of optogenetic manipulation in BALB/c nude mice bearing 4T1 cancer cells. (C), Coronal image co‐localization of ChR2‐mCherry with TH‐expressing neurons in VTA in BALB/c nude mice. Red: ChR2‐mCherry, green: TH‐positive neurons. Scale bar, 100 μm, insert scale bar 25 μm. (D), Percentage expression of TH and ChR2‐mCherry neurons in BALB/C‐Nu mice; n = 9 slices from 3 mice. (E), Representative example showing the distribution of ChR2‐mCherry‐expressing axons in the mPFC of a BALB/c nude mouse. Red: ChR2‐mCherry. Scale bar, 100 μm. (F), Tumor volume changes in 4T1 tumor‐bearing nude mice with ChR2 or mCherry with UCMS and chronic optogenetic stimulation; n = 9 control, n = 9 S‐mCherry, n = 11 S‐ChR2, ANOVA, Bonferroni post hoc test. (G) ~ (J), Serum concentrations of VEGF, bFGF, IL‐1a, and IL‐6; G, H, I, J n = 7 control, n = 7 S‐mCherry, H, J n = 7 S‐ChR2, G, I n = 6 S‐ChR2, ANOVA, Bonferroni post hoc test. *P < .05, **P < .01, ***P < .001

The experimental procedure is shown in Figure 5A‐B. Briefly, mice that expressed the virus cocktail (rAAV‐TH‐Cre and AAV‐DIO‐ChR2/mCherry) and subsequently underwent tumor implantation were then subjected to UCMS. However, at the end of the daily stress session, the VTA terminals in the mPFC of these mice were illuminated with blue light. Immunohistochemistry analysis showed robust ChR2 expression in the VTA of the nude mice (Figure 5C‐D). The expression of ChR2 was also found at the terminals of VTA^TH^ projections in the mPFC (Figure 5E). After chronic repetitive activation (10 Hz, 15 ms, 5‐minute ON/5‐min OFF, 30 minutes) of VTA^TH^ projections in the mPFC once every second day, tumor growth induced by stressors was significantly lower in the stress‐ChR2 group compared with the stress‐mCherry group, suggesting that blue‐light activation had an ameliorative effect (Figure 5F). Screening for tumor‐related cytokines, such as TNF‐a, VEGF, bFGF, IL‐1α, and IL‐6, as suggested by previous studies,^24^, ^25^, ^26^ was performed by an ELISA of collected serum. We found that VEGF, bFGF, and IL‐6 were higher under chronic stress than control mice, while the chronic repetitive activation of VTA^TH^ projections in the mPFC blocked stressor‐induced cytokine increase (Figure 5G‐I). Serum IL‐1α was significantly reduced in the stressed mice, and chronic repetitive activation of VTA^TH^ projections in the mPFC led to a significantly higher IL‐1α serum level than control mice and even higher than that of tumor‐bearing mice without stressors (Figure 5J). These data suggest that chronic light stimulation of VTA^TH^‐mPFC circuitry attenuated tumor growth promoted by chronic stressors.

Although dopamine D1 and D2 receptors both were expressed on cultured 4T1 breast tumor cells using immunohistochemistry (Figure S1B) and in 4T1 tumor tissue by fluorescence in situ hybridization (Figure S1A), there was no effect on the growth and proliferation of the cultured cells in vitro when these cells were treated with 0 to 4 μmol/L dopamine (Figure S1C).

### 3.6 Secretion of stress‐related hormones is correlated with 4T1 tumor size

**FIGURE 6:**
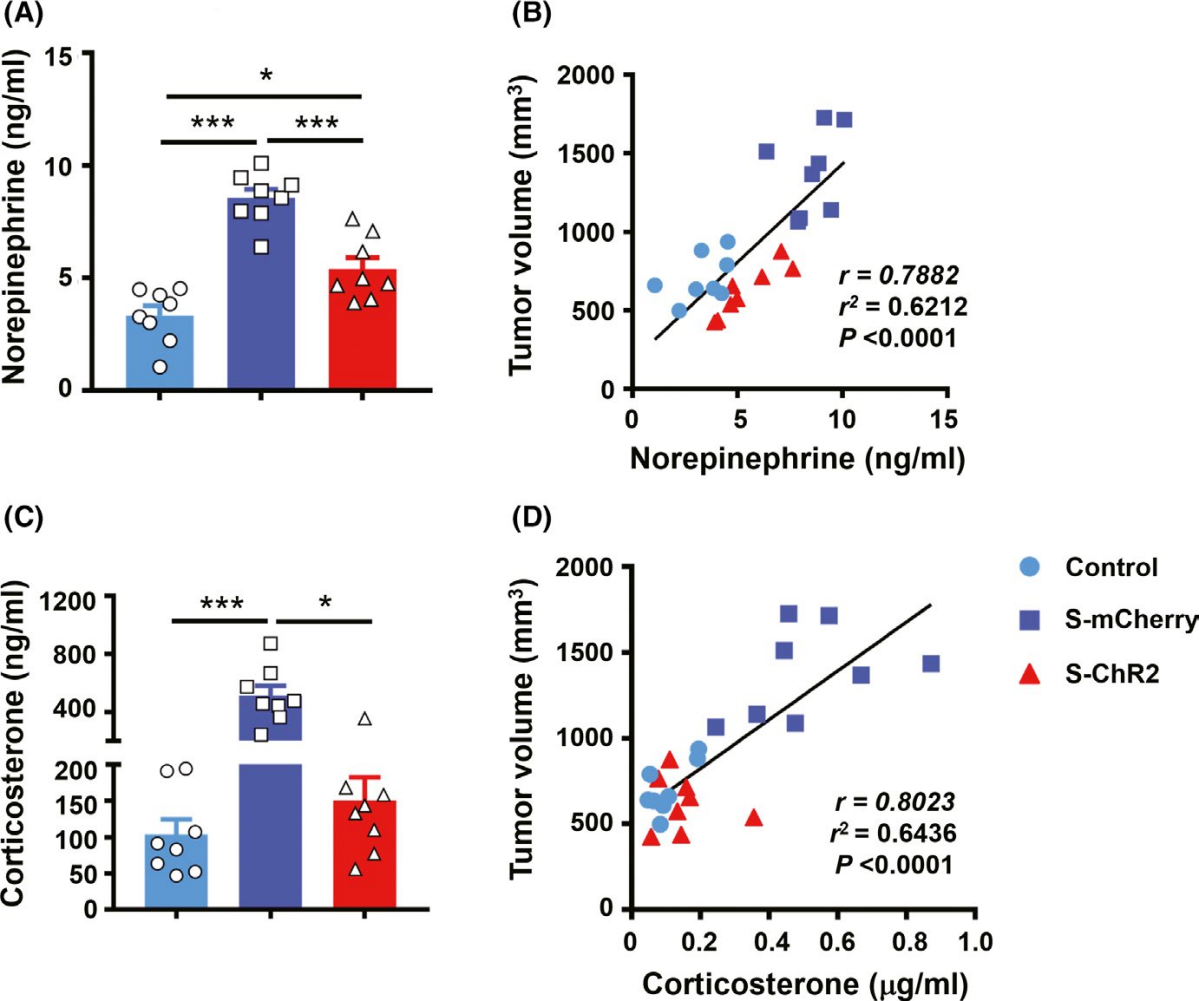
The secretion of stress‐related hormones and correlation with 4T1 tumor size. (A), Serum concentration of norepinephrine in stressed nude mice bearing 4T1 tumor cells; n = 8 mice/group, ANOVA, Bonferroni post hoc test. (B), Correlation between norepinephrine level and tumor volume for individual nude mice; n = 8 control, n = 8 s‐mCherry, n = 8 s‐ChR2, linear regression analysis, *P* < .0001, *r* ^2^ = .6212. (C), Serum levels of corticosterone in stressed nude mice bearing 4T1 tumor cells; n = 8 mice/group, ANOVA, Bonferroni post hoc test. (D), Correlation between corticosterone level and tumor volume in each mouse; n = 8 control, n = 8 s‐mCherry, n = 8 s‐ChR2, linear regression analysis, *P* < .0001, *r* ^2^ = .6436. *P < .05, **P < .01, ***P < .001

To establish a mechanistic link between neuromodulation and tumor growth, we investigated the impact of chronic repetitive activation of VTA dopaminergic terminals in the mPFC on neuroendocrine stress mediators, such as norepinephrine (NE) and corticosterone (CORT). Enzyme‐linked immunosorbent assay (ELISA) detected reduced NE (Figure 6A) and CORT (Figure 6C) levels in stressed mice expressing ChR2 following the chronic repetitive light stimulation upon VTA dopaminergic terminals in the mPFC compared with stressed mice expressing mCherry. Furthermore, the analysis of serum levels NE and CORT and tumor size showed that serum levels of NE and CORT were positively correlated with tumor volume across all groups (Figure 6B, NE, Pearson's *r* = .7882, *P* < .0001; Figure 6D, CORT, Pearson's *r* = .8023, *P* < .0001). This suggests that the link between the optic stimulation of DA terminals in the mPFC and reduction in anxiety induced by chronic stress and the reduction in tumor growth is correlative.

## 4 DISCUSSION

Breast cancer has the highest incidence rate of all cancers worldwide and has the second highest cancer death rate in the United States and fifth highest death rate worldwide.^28^, ^29^ Clinical studies have shown that, compared with the general population, cancer patients, and in particular, breast cancer and lung cancer patients, have a higher risk of anxiety and depression.^30^, ^31^ This may adversely affect cancer progression, outcome of treatment, and recovery in addition to quality of life and survival.^32^ Stress‐related disorders, such as anxiety and depression, can reprogram dendritic cells to suppress antitumor immune responses, resulting in poor efficacy of chemotherapy and immunotherapy,^33^ and the activation of the brain's reward system can modulate antitumor immunity.^34^ Thus, it is vital to investigate the intrinsic links between chronic stress, emotional state, and cancer progression. Here, we have shown that chronic stress promoted the progression of breast cancer in BLAB/c nude mice bearing MCF‐7 (Figure 3B‐C) and MDA‐MB‐231 (Figure 3D‐E). We demonstrated that repeated optogenetic stimulation of VTA^TH^ terminals within the mPFC, which had an anxiolytic effect (Figure 4F‐I), significantly attenuated tumor progression induced by chronic stress in BALB/c nude mice bearing 4T1. These findings have important clinical implications for the provision of new interventional and therapeutic strategies for the treatment of breast cancer.

In breast cancer studies, female mice are often used as model mice due to high incidences of breast cancer in females. Although the incidence of male breast cancer (MBC) is lower, male breast cancer is biologically similar to female breast cancer and the incidence of MBC is increasing.^35^, ^36^ In our study, we focused on the effects of neuronal regulation on cancer development under chronic stress. Of all cancer types, breast cancer patients have some of the highest levels of anxiety observed clinically.^30^ However, in studies related to stress or anxiety, male mice are often used as subjects because their endocrine levels are relatively stable in comparison with females, who have an estrous cycle. For example, one study based on canine inflammatory mammary cancer (IMC) and human inflammatory breast cancer (IBC) suggests that IBC and IMC male mouse models are a useful tool for modeling human inflammatory breast cancer.^37^ Similarly, our work (Figure 2) also showed that male mice bearing breast tumor cells were effective in our study.

Our bodies constantly adapt to both mental stress and physiological stress to sustain homeostasis during acute stress, without obvious lasting impact. However, chronic stress has many adverse effects on our body, including an increase in adiposity and eating behaviors that are detrimental to health. For example, evidence from rats indicates that reduced food intake and loss of bodyweight are criteria for stress severity.^38^ In humans, however, stress has a bidirectional effect on eating. Some individuals may eat less and lose weight during or after stress, and some may increase food intake and bodyweight during stress.^39^, ^40^ Studies using mice have shown that chronic stress combined with a high‐fat diet leads to significantly higher bodyweight than chronic stress with a standard diet. However, there was no difference in bodyweight between a chronic stress with standard diet group and a no stress with standard diet group.^22^ This is consistent with our results (Figure 1C‐D). However, we found that bodyweight decreased following daily UCMS in BALB/c nude mice bearing MCF‐7 (Figure 3C) and MDA‐MB‐231 (Figure 3E). This suggests that tumor growth and progression may affect bodyweight, whereas daily UCMS does not. In summary, these data indicate that daily UCMS over a period of four weeks promoted breast tumor growth and development (Figure 3).

Previous work shows that the circuitry from dopaminergic VTA neurons to medial prefrontal cortex (mPFC, one of largest projections from dopaminergic VTA neurons) is implicated in rewarding and aversive behavior, reinforcing processes and cognition function.^41^, ^42^, ^43^ Although photostimulation of lateral habenula (LHb) neurons that synapse mainly onto VTA dopamine neurons projecting to mPFC induces aversive behavior,^41^ and that excitation of the projection from dopaminergic VTA neurons onto the mPFC elicits positive/reinforcement behavior during reward,^42^ it remains unclear which type of neurons within the mPFC is innervated by the dopaminergic VTA neuron subset involved in reward/aversive behavior because of the complexity of dopaminergic VTA neuronal subsets and functions.^44^ Neither is it known what role VTA DA‐mPFC circuitry plays during or after stress. Much evidence, however, shows that the brain‐reward system is related to stress. For example, the mesocortical DA system can be selectively activated by stress^45^; optogenetic activation of VTA DA neurons regulates depression‐related behavior induced by chronic mild stress^46^ and optical inhibition of the VTA‐mPFC pathway by activation of NpHR induces susceptible phenotype of depressive‐like behavior.^47^ In addition, microdialysis experiments demonstrate a marked increase in extracellular dopamine within the mPFC and other brain regions in response to restraint stress,^48^ which may provide evidence for the hypothesis that VTA DA neurons are activated during or after stress to provide an antistress role by generating a hedonic impact due to the effect of rewards.^49^ Furthermore, optical activation of D1 cells in the mPFC by ChR2 in mice has an antidepressant and anxiolytic effect and infusion of D1 receptor agonist into the mPFC also produce antidepressant and anxiolytic responses.^12^ In accordance with this evidence, we hypothesize that the activation of the projection from VTA DA into the mPFC has an antistress/anxiolytic effect. Here, we demonstrate that specific activation of the terminals within the mPFC from VTA TH neurons by optogenetic manipulation in mice rescues anxiety‐like behavior induced by chronic stress (Figure 4). This suggests that the VTA TH‐mPFC circuitry has an antistress/anxiolytic role.

It has long been known that the effects of stress on the body's immune function are like that of a double‐edged sword.^50^, ^51^ Although acute stress may have a positive effect for the organism,^52^ chronic stress is generally adverse and leads to serious health complications, including dysfunction of immunity.^5^, ^25^, ^33^, ^53^, ^54^ In our work, we found that serum VEGF, bFGF, and IL‐6, which are associated with tumor growth and progression,^25^, ^27^ were increased (Figure 5G‐H and J) and IL‐1a, which promotes antitumor immune effects,^26^ and was lower in BALB/c nude mice bearing 4T1 after chronic stress (Figure 5I). Chronic repetitive activation of VTA^TH^ projections in the mPFC can rescue these effects induced by chronic stress. This is consistent with the trend for change in tumor volume (Figure 5F). Moreover, there is evidence that VEGF and IL‐6 are upregulated by norepinephrine (NE), a hormone that rises in response to the stress^55^ in cultured tumor cells.^25^ In the present study, we found that NE was higher after chronic stress than without stress (Figure 2).

Administration of dopamine can ameliorate tumor growth and progression by inhibiting angiogenesis in a nude mice model of gastric cancer.^56^ However, an in vitro experiment suggests that dopamine has no direct effect on the survival and proliferation of tumor cells.^57^ This is consistent with our result showing no difference in the survival and proliferation of breast tumor cells treated with different concentrations of dopamine (Figure S1). This implies that the activation of dopaminergic neural circuits may affect tumor growth through mechanisms other than affecting tumor cells directly. The more detailed mechanisms underlying the role of dopaminergic neural circuitry in tumor growth require further investigation.

In summary, we have demonstrated that specific activation of VTA^TH^ terminals within the mPFC by optogenetic manipulation ameliorated stress‐induced anxiety‐like behavior, and furthermore, we found that chronic repetitive optogenetic stimulation of VTA^TH^ inputs to the mPFC markedly attenuated stress‐induced progression of breast cancer in BALB/c nude mice. These results strongly suggest an impact of emotional status on tumor growth. These findings reveal a link between stress, regulation of emotion, and progression of breast cancer, and provide new insight on therapeutic interventions in breast cancer treatment.

## CONFLICT OF INTEREST

The authors claim that there are no conflicts of interest.

## AUTHOR CONTRIBUTIONS

JT conceived the study. X‐RX, Q.X, Y‐C.H, and X‐Y. Z. performed experiments. YL advised regularly. JT and X‐R.X analyzed data. JT and X‐RX wrote the manuscript.

## Supporting information

Fig S1

## Data Availability

The data that support the findings of this study are available from the corresponding author upon reasonable request (jie.tu@siat.ac.cn).
