# Supplementary material for: Activation of dopaminergic VTA inputs to the mPFC ameliorates chronic stress‐induced breast tumor progression
Source: CNS Neurosci Ther. 2020 Oct 28;27(2):206–19. doi: 10.1111/cns.13465 (PMC7816210; doi:10.1111/cns.13465)
Supplement: Supplementary file 1 — Fig S1 [file CNS-27-206-s001.docx]

**Supplementary file**

**
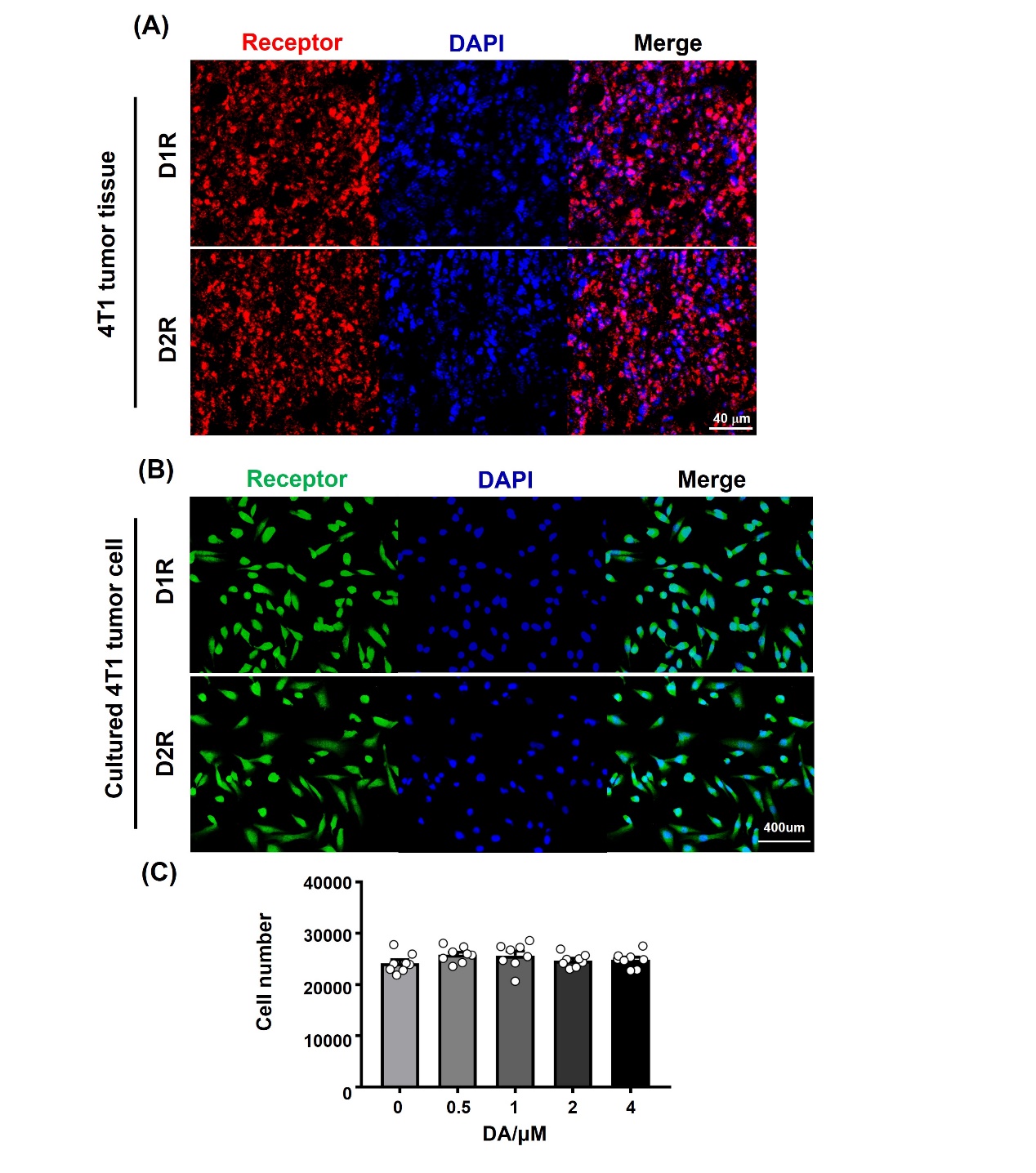
**

**Supplementary FIGURE 1** Proliferation of 4T1 tumor cells are not affected by dopamine administration *in vitro.* (A), Representative images of D1 (upper) and D2 (lower) receptors expressed in tumor tissue. Red: receptors, blue: DAPI. (B), Representative images of D1 (upper) and D2 (lower) receptors expressed in cultured 4T1 cells. Green: receptors, blue: DAPI. (C), Proliferation of 4T1 cells treated by different concentrations of dopamine (0, 0.5, 1, 2 and 4 μM); n=8 wells/group, ANOVA, Bonferroni post-hoc test.
